# Autonomic and brain responses associated with empathy deficits in autism spectrum disorder

**DOI:** 10.1002/hbm.22840

**Published:** 2015-05-21

**Authors:** Xiaosi Gu, Tehila Eilam‐Stock, Thomas Zhou, Evdokia Anagnostou, Alexander Kolevzon, Latha Soorya, Patrick R. Hof, Karl J. Friston, Jin Fan

**Affiliations:** ^1^ Wellcome Trust Centre for Neuroimaging, University College London London United Kingdom; ^2^ Virignia Tech Carilion Research Institute Roanoke Virignia; ^3^ Department of Psychology Queens College, The City University of New York Flushing New York; ^4^ The Graduate Center The City University of New York New York New York; ^5^ Department of Psychiatry Icahn School of Medicine at Mount Sinai New York New York; ^6^ Bloorview Research Institute University of Toronto Toronto Canada; ^7^ Seaver Autism Center for Research and Treatment Icahn School of Medicine at Mount Sinai New York New York; ^8^ Rush University Chicago; ^9^ Fishberg Department of Neuroscience Icahn School of Medicine at Mount Sinai New York New York; ^10^ Friedman Brain Institute Icahn School of Medicine at Mount Sinai New York New York

**Keywords:** autism spectrum disorder, autonomic nervous system, functional magnetic resonance imaging, brain connectivity, empathy, dynamic causal modeling, interoceptive inference

## Abstract

Accumulating evidence suggests that autonomic signals and their cortical representations are closely linked to emotional processes, and that related abnormalities could lead to social deficits. Although socio‐emotional impairments are a defining feature of autism spectrum disorder (ASD), empirical evidence directly supporting the link between autonomic, cortical, and socio‐emotional abnormalities in ASD is still lacking. In this study, we examined autonomic arousal indexed by skin conductance responses (SCR), concurrent cortical responses measured by functional magnetic resonance imaging, and effective brain connectivity estimated by dynamic causal modeling in seventeen unmedicated high‐functioning adults with ASD and seventeen matched controls while they performed an empathy‐for‐pain task. Compared to controls, adults with ASD showed enhanced SCR related to empathetic pain, along with increased neural activity in the anterior insular cortex, although their behavioral empathetic pain discriminability was reduced and overall SCR was decreased. ASD individuals also showed enhanced correlation between SCR and neural activities in the anterior insular cortex. Importantly, significant group differences in effective brain connectivity were limited to greater reduction in the negative intrinsic connectivity of the anterior insular cortex in the ASD group, indicating a failure in attenuating anterior insular responses to empathetic pain. These results suggest that aberrant interoceptive precision, as indexed by abnormalities in autonomic activity and its central representations, may underlie empathy deficits in ASD. *Hum Brain Mapp 36:3323–3338, 2015*. © **2015 The Authors Human Brain Mapping Published byWiley Periodicals, Inc.**

## Introduction

Autism spectrum disorder (ASD) is a family of neurodevelopmental disorders with a wide range of sensory and socio‐emotional deficits [Chiu et al., 2008; Dinstein et al., 2012; Happe et al., 2006]. Empathy, the ability to share vicariously the feelings of others, is an important social‐emotional faculty [Gu et al., 2010; Moriguchi et al., 2007] and is compromised in individuals with ASD [Baron‐Cohen and Wheelwright, 2004]. Empathy is considered a multifaceted construct, including at least emotional contagion and arousal and cognitive perspective‐taking [de Waal, 2008]. Previous studies have demonstrated abnormalities in various aspects of empathy in individuals with ASD, including difficulties in mentalizing and perspective‐taking [Fan et al., 2014; Hadjikhani et al., 2014; Minio‐Paluello et al., 2009], as well as heightened affective arousal to emotional stimuli [Fan et al., 2014; Smith, 2009]. However, there has not been a mechanistic account for these socio‐emotional deficits in ASD.

It has been proposed that a core component of empathy is the mechanism through which the observer gains access to the subjective state of another person via the observer's own neural and bodily representations [Decety and Jackson, 2004; Preston and de Waal, 2002; Singer et al., 2009]. The anterior insular cortex (AIC) and its associated autonomic processing are considered to be crucial in supporting this embodied or interoceptive “theory of mind” [Corradi‐Dell'Acqua et al., 2011; Craig, 2014; Gu et al., 2010, 2012; Singer et al., 2009; Wicker et al., 2003]. The AIC is a critical cortical center in the interoceptive system which processes information from the body and exerts autonomic control [Craig, 2009; Craig, 2011; Critchley and Harrison, 2013; Gu et al., 2013a]. For instance, a direct correlation has been found between autonomic activity indexed by skin conductance response (SCR) and neural activity in the AIC measured by functional magnetic resonance imaging (fMRI) during resting state in neurotypical individuals [Eilam‐Stock et al., 2014; Fan et al., 2012]. Using fMRI [Gu and Han, 2007; Gu et al., 2010, 2013b], activation likelihood estimate meta‐analysis and neuropsychological approaches [Gu et al., 2012], we previously demonstrated that the AIC is specifically activated during, and is necessary for, empathetic pain processing. Importantly, we showed that even when the participant's attention was directed away from the painful aspect of images depicting another person's pain, the AIC was still more activated for painful compared to neutral stimuli, while the anterior cingulate cortex showed comparable activations for painful and neutral stimuli [Gu et al., 2010]. Moreover, accumulating evidence suggests that autonomic signals and their higher‐order rerepresentations are crucial for emotional feelings [Craig, 2002; Critchley and Harrison, 2013; Ekman et al., 1983; Gray and Critchley, 2007; Harrison et al., 2010; Rainville et al., 2006].

Several other brain regions encoding biological information are also involved in social and emotional processes [Saxe, 2006]. The extrastriate body area (EBA) is involved in the exteroceptive processing of visual features related to the body during empathetic responses [Lamm and Decety, 2008]. Although much attention has been devoted to general visual deficits in ASD [Behrmann et al., 2006; Dakin and Frith, 2005; Kaiser et al., 2010], little is known about the involvement of EBA in ASD. The prefrontal cortex (PFC), especially the lateral PFC (LPFC), has been associated with executive control and information integration during socio‐emotional processing, and is a domain‐general area [Corbetta and Shulman, 2002; Levy and Wagner, 2011; Romanski, 2007]. Deficits in LPFC responses have been found in individuals with ASD [Kaiser et al., 2010; Shafritz et al., 2008; Silk et al., 2006], supporting the hypothesis that ASD individuals have difficulty in integrating information from different modalities [Happe and Frith, 2006].

Considering the complex nature of socio‐emotional functions and the manifestation of abnormalities at both sensory and socio‐emotional levels in ASD, it is important to review normative accounts of the disorder. Several recent articles have proposed such models of ASD [Friston et al., 2013b; Lawson et al., 2014; Pellicano and Burr, 2012; Quattrocki and Friston, 2014; Van de Cruys et al., 2014) based on the notion that the brain uses generative models of the world to actively infer the causes of sensory input to predict appropriate (expected) visceral and motor responses [Friston, 2010; Friston et al., 2013a]. In this setting, the influence of these prediction errors is nuanced by their expected *precision*. Computationally, precision corresponds to reliability or inverse variability. Psychologically, precision can be regarded as the attention paid to sensory channels [Feldman and Friston, 2010]. Physiologically, this precision or attention is thought to be mediated by the postsynaptic gain or sensitivity of neuronal populations reporting prediction error [Bastos et al., 2012]. The specific failure in ASD has been attributed to a relative increase in the precision of sensory evidence over the precision of higher (extrasensory) beliefs [Friston et al., 2013b; Lawson et al., 2014; Pellicano and Burr, 2012; Quattrocki and Friston, 2014; Van de Cruys et al., 2014].

Crucially, it has been hypothesized that aberrantly high precision in the interoceptive domain might account for selective socio‐emotional deficits in ASD [Friston et al., 2014; Van de Cruys et al., 2014], given the intimate relationship between autonomic activity, their related cortical responses, and socio‐emotional awareness. These proposals provide a useful framework for a quantitative and mechanistic understanding of socio‐emotional deficits in ASD in terms of failures in Bayesian inference, leading to false inference about interoceptive and emotional states, particularly in the context of prosocial and affiliative interactions. Based on these proposals and empirical findings on interoception, we have recently proposed that the AIC integrates bottom‐up interoceptive signals with top‐down predictions to generate a representation or expectation about embodied states [Gu et al., 2013a]. This mechanism enables the AIC to contextualize descending predictions to visceral systems that provide a point of reference for autonomic reflexes. This process has been termed *interoceptive inference*, namely, Bayesian inference about interoceptive states [Gu and FitzGerald, 2014; Seth, 2013; Seth et al., 2011]. Empirically, it remained unclear how deficits in interoceptive inference directly contribute to socio‐emotional deficits in ASD.

We hypothesized that individuals with ASD would show abnormally high interoceptive precision during empathetic pain processing, considering previous findings of increased autonomic activities [Hirstein et al., 2001; Kylliainen and Hietanen, 2006; Van Hecke et al., 2009] and heightened emotional arousal in ASD [Fan et al., 2014] during socio‐emotional processing. To test this hypothesis, we used simultaneous SCR and fMRI measures during a well‐validated empathy‐for‐pain paradigm [Gu et al., 2012; Gu et al., 2010] in high‐functioning male adults with ASD and matched healthy controls (HC). Importantly, we modeled *interoceptive precision* in terms of the modulatory effect exerted by experimental context (i.e. viewing others' pain) on the within‐area self‐connection of AIC using dynamic causal modeling (DCM) [Friston et al., 2003; Penny et al., 2004]. The self‐connection of a given neural region is assumed to be negative so that its activity returns to equilibrium levels; thereby modeling cortical gain control. Experimentally induced increases of gain are modeled as an attenuation of self‐inhibition—that effectively increases the excitability of neuronal populations (i.e., disinhibition). Therefore, changes in self‐disinhibition reflect changes in gain (or precision) following experimental manipulations. Using DCM, we also modeled the directed interactions among the LPFC, AIC, and EBA, and estimated how experimental context modulates directed connections among these cortical areas [Friston et al., 2003; Penny et al., 2004; Stephan et al., 2010] to test a competing hypothesis that decreased precision at the higher level of LPFC and decreased top‐down connectivity from the LPFC to AIC, rather than increased interoceptive precision, contributes to empathy deficits in ASD. Our hypothesis makes a number of specific predictions: individuals with ASD would show (1) disinhibited (peripheral) autonomic responses to arousing empathetic pain stimuli; (2) disinhibited or increased cortical response to empathetic pain in brain regions subserving interoceptive and autonomic processes, such as the AIC; and (3) greater modulation of self‐connectivity within the AIC by empathetic pain.

## Materials and Methods

### Participants

We recruited 17 unmedicated high‐functioning adult males with ASD and 18 matched HC participants through the Seaver Autism Center for Research and Treatment at the Icahn School of Medicine at Mount Sinai (ISMMS). One HC participant was excluded due to chance‐level behavioral performance on the empathy‐for‐pain paradigm, resulting in a final sample of 17 participants in each group (Table 1). One additional HC participant had incomplete SCR data and was therefore excluded from the SCR analysis, yielding *n* = 17 for ASD and *n* = 16 for HC for the SCR results. Two ASD and two HC participants were excluded from the fMRI analysis due to excessive head motion, yielding 15 participants in each group for the fMRI results. One of the HC participants, who was excluded due to motion, also did not complete the self‐report questionnaires. Individuals in the ASD group met diagnostic criteria for autism disorder (*n* = 12) or Asperger syndrome (*n* = 5) by psychiatric interview according to the Diagnostic and Statistical Manual‐IV (DSM‐IV‐TR) [Association, 2000], confirmed by the Autism Diagnostic Interview‐Revised (ADI‐R; [Lord et al., 1994]) and Autism Diagnostic Observation Schedule‐Generic (ADOS‐G; [Lord et al., 2000]), except for one participant for whom ADI‐R scores were unavailable. Participants who met criteria only for Pervasive Developmental Disorder not Otherwise Specified (PDD‐NOS) by DSM‐IV‐TR were excluded. Other exclusion criteria included epilepsy, history of schizophrenia, schizoaffective disorder or other Axis I mental disorders except obsessive‐compulsive disorder (given the phenotypic overlap with ASD), and use of depot neuroleptic medication or other psychoactive drugs within five weeks prior to participation. For the HC group, exclusion criteria were medical illness or history in first‐degree relatives of developmental disorders, learning disabilities, autism, affective disorders, and anxiety disorders. Participants from both groups with a history of substance or alcohol dependency or abuse within 1 year prior to participation were excluded as well. Each group had 16 right‐handed and 1 left‐handed participants (measured by the Edinburgh Inventory Handedness Questionnaire [Oldfield, 1971]. HCs were matched with ASD participants for age, parental and participants' socioeconomic status (SES), and Full‐Scale Intelligence Quotient (FSIQ) measured with the Wechsler Adult Intelligence Scale [WAIS‐III; [Wechsler, 1997]], and had an FSIQ in the low average range or higher (>80). All participants provided written informed consent, approved by the ISMMS Institutional Review Board.

**Table 1:** Demographic data

	ASD (*n* = 17)	HC (*n* = 17)	*P*
Age (years)	26.2 ± 6.4	26.8 ± 7.8	>0.7
Handedness score	73.5 ± 35.3	75.6 ± 40.5	>0.8
Parental SES^a^	91.3 ± 16.8	92.0 ± 22.6	>0.9
Subject SES^a^	27.9 ± 14.6	32.7 ± 15.4	>0.3
Full Scale IQ	109.5 ± 18.0	113.5 ± 11.9	>0.4
ASD diagnosis (autism/Asperger)	12/5		
ADI‐R^b^			
Social	18.6 ± 8.0		
Verbal communication	15.5 ± 4.9		
Repetitive behavior	6.2 ± 3.7		
Development	3.5 ± 1.5		
ADOS‐G^c^			
Communication	3.1± 1.3		
Social	6.9 ± 2.1		
Imagination	0.7 ± 0.5		
Stereotyped behaviors	1.3 ± 1.4		

a SES data was not available for one ASD participant and one HC participant.

b ADI‐R scores were not available for one ASD participant. ASD: autism spectrum disorder; HC: healthy control. Data are reported as means ± standard deviation.

### Behavioral paradigm

**Figure 1:**
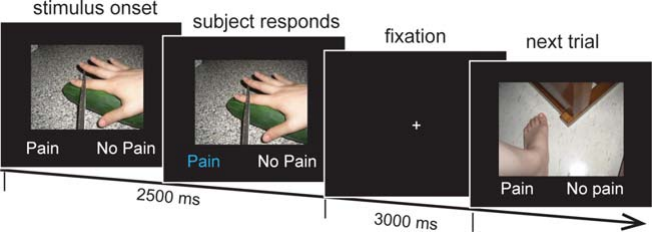
Empathy‐for‐pain paradigm. Participants viewed images of others in painful and nonpainful situations and indicated whether the person in the image was suffering from pain.

Participants were presented with 256 color photographs of hands or feet of individuals in painful or nonpainful situations (Fig. 1), and were asked to judge whether the person shown in the image was suffering from pain or not. As in our previous studies [Gu et al., 2012, 2010, 2013b], these photos depicted everyday life scenarios and were taken from a first‐person perspective to avoid mental rotation. Half of the pictures showed painful situations and the other half showed nonpainful scenarios that were identical in terms of physical properties such as brightness and contrast. All images were slightly blurred with a Gaussian filter to remove gender or age related information.

Sixty‐four images were presented in each run, for a total of four runs. Stimuli were presented in an event‐related fMRI design and presentation of each type of picture (8 types for laterality: left/right, body part: hand/foot, and pain: painful/nonpainful) were counterbalanced in a Latin Square (pseudo‐randomized) design with all types of picture proceeded and followed by other types with an equal probability. There was a 30‐s fixation period at the beginning and end of each run to allow skin conductance and hemodynamic responses to return to baseline. It has been shown that 30‐s is sufficient for both the skin conductance response [Bach et al., 2010] and haemodynamic response [Friston et al., 1995] to return to baseline. This reflects the formal similarity between the impulse response function for SCR and the hemodynamic response function (HRF) [Bach et al., 2010]. Each 5.5‐s trial consisted of a picture presentation and two response options (i.e., no pain vs. pain) for 2.5‐s. The participants were asked to make responses within the 2.5‐s time window. This was followed by 3‐s of fixation. Participants made button‐press responses with their right hand.

### Behavioral data analysis

We analyzed behavioral accuracy data using signal detection theory (SDT) [Snodgrass and Corwin, 1988]. SDT is a method that discerns signal from noise, and assumes that the perceiver has a distribution of internal responses for both signal and noise. Participants' sensitivity to signals is calculated as *d*′ = (*μ*_s_ – *μ*_n_)/sd, where *μ*_s_ is the mean of the signal (pain) distribution, *μ*_n_ is the mean of the noise (no pain) distribution, and sd is the common standard deviation of both distributions. A larger *d*′ represents better discrimination accuracy and a smaller *d*′ denotes poorer discriminability. Decision bias *β* was calculated as *β* = *f*_s_(λ)/*f*_n_(λ), where *f*_s_(λ) is the height of the signal distribution at a given criterion λ and *f*_n_(λ) is the height of the noise distribution at the same λ.

### Trait assessments

All participants completed personality assessments of trait alexithymia and trait empathy. Trait alexithymia was measured using the 20‐item Toronto Alexithymia Scale (TAS‐20) [Bagby et al., 1994]; higher scores indicate greater difficulty in emotional awareness and greater degree of alexithymia. Trait empathy was measured using the Empathy Quotient (EQ), a 40‐item self‐report questionnaire without subscales [Baron‐Cohen and Wheelwright, 2004]; higher scores indicate greater trait empathy.

### Statistical comparisons

Because group comparisons were based on *a priori* hypotheses in small samples, we used the nonparametric bootstrapping method [Hasson et al., 2003; Mooney, 1993] for the behavioral, SCR, and DCM connectivity parameters to assess the probability of observing a difference between two groups (*n*_1_ participants for the HC group and *n*_2_ participants for the ASD group) by chance. The bootstrapping procedure was conducted with 10,000 iterations as follows: (1) *n*_1_ participants were selected randomly as the surrogate HC group, from the whole group of (*n*_1_ *+ n*_2_) participants including both ASD and HC participants; (2) *n*_2_ participants were selected randomly as the surrogate ASD group from the whole group of (*n*_1_ *+ n*_2_) participants; and (3) the *t* value of the difference between the two surrogate groups was calculated. After 10,000 iterations, the distribution of the *t* values was obtained. The observed *t* value (i.e., between ASD and HC groups) was then calculated and compared along the *t* distribution. If the probability of obtaining the observed *t* value along the permutated distribution of *t* value was less than 5%, we considered the difference between the ASD and HC groups to be significant. For correlations, we calculated Pearson correlation coefficients and statistical significance was set at *P* = 0.05, two‐tailed.

### Skin conductance acquisition and analysis

Skin conductance was acquired during fMRI scanning as described in our previous study [Fan et al., 2012]. In brief, skin conductance was recorded using the GSR100C amplifier (BIOPAC Systems, Goleta, CA), together with the base module MP150 and the AcqKnowledge software (version 3.9.1.6). The GSR100C measures skin conductance by applying a constant voltage of 0.5 V between two electrodes that are attached to the palmar skin. Skin conductance (measured in μS) was recorded using a 2000‐Hz sampling rate (gain = 2 μS/V, both high pass filters = DC, low pass filter = 10 Hz). After cleaning the skin with alcohol preps, two EL507 disposable EDA (isotonic gel) electrodes were placed on the palmar surface of the distal phalanges of the big and second toes of left foot. The signal was low‐pass filtered (using the MRI‐Compatible MRI CBL/FILTER System MECMRI‐TRANS) to reduce radio frequency interference from the scanner. BIOPAC recording was synchronized to the E‐Prime program via the parallel port of the computers. Event markers were recorded to enable precise time alignment of skin conductance recording with scan onsets and task trials.

To analyze the SCR data, we applied general linear modeling (GLM) using SCRalyze v.b2.1.7 [Bach et al., 2010]. SCR data were epoched into individual runs, and the range of trimming was determined by the beginning of the first marker and the end of the last marker of each block with 30‐s before and after the first and last marker for baseline. Consistent with fMRI analysis (see fMRI methods below), the two vectors for onsets of the events (“all images” and “painful images”) in seconds were extracted based on the corresponding markers recorded. The regressors were then generated by convolving the vectors with the canonical response function of the SCR [Bach et al., 2010]. GLM was then performed with a band‐pass filter (first‐order Butterworth filter) with a band‐pass filter of 0.01‐0.12 Hz, similar to our previous study on SCR [Fan et al., 2012]. The data were then normalized to control for between‐subject differences in skin conductance response amplitude. The *β* values (nondimensional) corresponding to the two regressors were obtained from the GLM of each participant for between‐group statistical testing.

We also extracted single trial SCRs associated with the presentation of each stimulus by modeling each trial as a separate regressor and “all other images” and “all other painful images” as two further regressors, iterated over 64 trials and over 4 sessions. These trial‐to‐trial SCR parameter estimates were later used as a parametric modulatory regressor for an fMRI analysis to test for trial‐by‐trial correlations between SCR and fMRI responses (see fMRI methods).

### fMRI data acquisition and preprocessing

All MRI acquisitions were obtained on a 3‐T Siemens Allegra MRI system at ISMMS. All participants underwent only one session with all scanning sequences. The whole scan session lasted about 1.5‐h. Foam padding was used to keep participants' heads still. All images were acquired along axial planes parallel to the anterior commissure‐posterior commissure (AC‐PC) line. A high‐resolution T2‐weighted anatomical volume of the whole brain were acquired on an axial plane parallel to the AC‐PC line, with a turbo spin‐echo (TSE) pulse sequence with the following parameters: 40 axial 4 mm‐thick slices, skip = 0 mm, repetition time (TR) = 4050 ms, echo time (TE) = 99 ms, flip angle = 170°, field of view (FOV) = 240 mm, matrix size = 448 × 512, voxel size = 0.47 × 0.47 × 4 mm. T2*‐weighted images were acquired for fMRI. Slices were obtained corresponding to the T2‐weighted images. The fMRI imaging was performed using a gradient echoplanar imaging (GE‐EPI) sequence: 40 axial slices, 4 mm‐thick, and skip = 0 mm, TR = 2500 ms, TE = 27 ms, flip angle = 82°, FOV = 240 mm, and matrix size = 64 × 64. Each run started with two dummy volumes before the onset of the task to allow for equilibration of T1 saturation effects. A total of four EPI runs with 165 image volumes per run were acquired for each participant. Event‐related analyses of the fMRI data from the task were conducted using statistical parametric mapping (SPM8; Wellcome Department of Imaging Neuroscience, London, UK). The functional images were adjusted for slice timing, realigned to the first volume, coregistered to the T2 image, normalized to a standard template (MNI, Montreal Neurological Institute), resampled to a 2 × 2 × 2 mm voxel size, and spatially smoothed with an 8 × 8 × 8 mm full‐width‐at‐half‐maximum (FWHM) Gaussian kernel.

### General linear modeling of fMRI data

For the main fMRI analysis presented in Fig. 4A, we included two task events of interest; onset of “all images” and “painful images”, in first‐level GLM [Friston et al., 1995]. In this model specification, parameter estimates of “painful images” represent brain activations related to empathetic pain that is over and above the activation related to “all images”. This can be considered as equivalent to the response to the “non‐painful” images. We used this model specification to specify unique driving and modulatory inputs for DCM (see below). The responses to “painful images” in this setting are equivalent to the activations related to “painful” minus “non‐painful” from the mathematically equivalent model where “painful images” and “non‐painful images” are modeled separately (see Supporting Information Fig. S1). For the analysis on SCR‐fMRI correlation presented in Fig. 4B, we constructed an additional GLM where trial‐by‐trial parameter estimates of SCR were entered as parametric modulators at the onset of stimulus presentation.

GLM was conducted for the functional scans from each participant by modeling the observed event‐related BOLD signals and regressors to identify the relationship between the task events and the hemodynamic response. Regressors were created by convolving a train of delta functions representing the sequence of individual events with the default SPM basis function [Friston et al., 1998]. Six parameters generated during motion correction were entered as covariates. First‐level SPMs from all participants were entered into a second‐level between‐group analysis. We used the Monte Carlo method to determine the statistical threshold [Slotnick et al., 2003]. The general idea is to model the whole brain volume of 64 × 64 × 40 original voxels, assume type I error of an individual voxel at *P* < 0.05, smooth the volume with a 3‐dimensional 8‐mm FWHM Gaussian kernel, and then count the size of each contiguous cluster of voxels. After 1000 iterations, a probability associated with each cluster extent is calculated across all iterations, and a cluster extent can be chosen to achieve the desired correction for multiple comparisons. Assuming an individual voxel type I error of *P* < 0.05, a cluster extent of 120 contiguous resampled voxels (2 × 2 × 2 mm^3^) was indicated as necessary to correct for multiple comparisons at *P* < 0.05 (see Supporting Information Fig. S2). This same threshold was applied to all contrasts.

For the analysis of brain–behavior relationship, we quantified BOLD responses to viewing painful images in our main regions of interest (ROIs), namely, AIC, EBA, and LPFC, based on the peak activations averaged over both groups as listed in Table 3 (5‐mm spherical ROI centered at [−38, 20, 2] for AIC, [−46, −70, −4] for EBA, and [58,14,22] for LPFC]). These parameter estimates were then correlated with behavioral measures, such as the behavioral sensitivity index *d*' and trait measures of EQ and TAS‐20.

### Dynamic causal modeling

DCM uses a deterministic model of neural dynamics in a network of interacting brain areas to provide Bayesian estimates of the effective strength of synaptic connections among neuronal populations and modulatory effect of experimental manipulations as well as model evidence [Friston et al., 2003; Penny et al., 2004]. Specifically, DCM uses a differential equation $\dot{x}=\left(A+uB\right)x+Cu$ where *x* is a vector of the neuronal states summarizing the activity of regions of interests (ROIs) and u is a vector representing external or experimental input. Please see Supporting Information Fig S3 for a simplified illustration of the general idea behind this model. The A matrix includes the strength of fixed connections between ROIs; that is, connections that are not modulated by experimental input. The B matrix represents the degree of the modulation of these connections induced by the experimental input; that is, painful stimuli or “modulatory input.” Matrix C is the direct influence on the ROI usually attributed to sensory input (i.e., EBA directly receives visual body information; i.e., all stimuli or “driving input”). These results, therefore, are generative models of the brain that provide Bayesian posterior estimates of the effective strength of synaptic connections among neuronal populations and modulatory or contextual effect of experimental manipulations [Friston et al., 2003; Penny et al., 2004]. DCM also allows one to define models with different network properties, and then select the best model or the best family of models using Bayesian model comparison [Stephan et al., 2009, 2010].

#### Model specification

DCM was implemented using SPM (SPM12; Wellcome Department of Imaging Neuroscience, London, UK). Our three‐region DCM was motivated by the main results of the fMRI GLM analysis (see Fig. 4A and Table 3) showing group differences in the AIC, EBA, and LPFC. However, the selection of ROI coordinates was based on the group average, rather than group difference, related to empathetic pain. The AIC is our key ROI and is important for interoceptive processing of emotional stimuli [Gu et al., 2013a]. The EBA is involved in (exteroceptive) processing of visual features of body parts, as reviewed in the Introduction [Lamm and Decety, 2008]. The EBA was included to contrast with the interoceptive AIC pathway but also needed as the node for visual input (i.e., “driving input”). The LPFC is involved in general cognitive control and executive functions [Corbetta and Shulman, 2002; Levy and Wagner, 2011]. Although they are rather minimal, these models have the requisite hierarchical structure: two cortical levels (high: LPFC; low: AIC and EBA) and two pathways (interoceptive: LPFC‐AIC; exteroceptive: LPFC‐EBA). Only unilateral ROIs were included because—for both AIC and LPFC—we only detected unilateral activation related to painful images across both groups; and for all three ROIs, group differences in pain‐related activations were limited to one hemisphere. We assumed reciprocal (extrinsic) connections among all three regions as well as recurrent self (intrinsic) connections within all three regions. Image viewing served as a driving input to EBA in all models. Viewing painful images served as modulatory input changing either no intrinsic connections (models 1–4), or intrinsic connections in AIC, EBA, and LPFC (models 5–8). Additionally, painful images could modulate either no connection between AIC and EBA, and LPFC (models 1 and 5), forward connections from AIC and EBA to LPFC (models 2 and 6), backward connections from LPFC to AIC and EBA (models 3 and 7), or reciprocal connections between AIC and EBA, and LPFC (model 4 and 8; see Fig. 5 for the 8 models).

#### Time series extraction

We created volumes of interest (VOIs) of 8‐mm radius based on each participant's local maximum of empathetic pain‐related activation closest to group mean: AIC, centered at [−38 20 2]); EBA, centered at [−46 −70 −4]); and LPFC, centered at [58 14 22] (see Table 3). We then extracted fMRI time series from individual VOIs using their principal eigenvariates. One HC participant failed to display activation in these three VOIs, even at *P* < 0.1 uncorrected and was therefore excluded from the analysis.

#### Bayesian model selection

DCMs were estimated at the within‐subject or individual level first. Each participant had one DCM per model per session. For the same model, four DCMs for the four sessions were averaged within‐subject using Bayesian model averaging [Penny et al., 2010] in a parameter‐specific fashion, to allow for later statistical comparisons. We then conducted Bayesian model selection (BMS) among all models at both the model level and the family level (with or without changes in intrinsic connections). Model inference was made at the group level using random effects. The model/family with the highest excedance probability was selected as the optimal model/family. Group differences in the parameters of the optimal model were tested with the bootstrapping method as described before. The Bonferroni procedure was used to correct for multiple comparisons [Dunn, 1961].

## Results

### Behavioral and trait measurements

**Figure 2:**
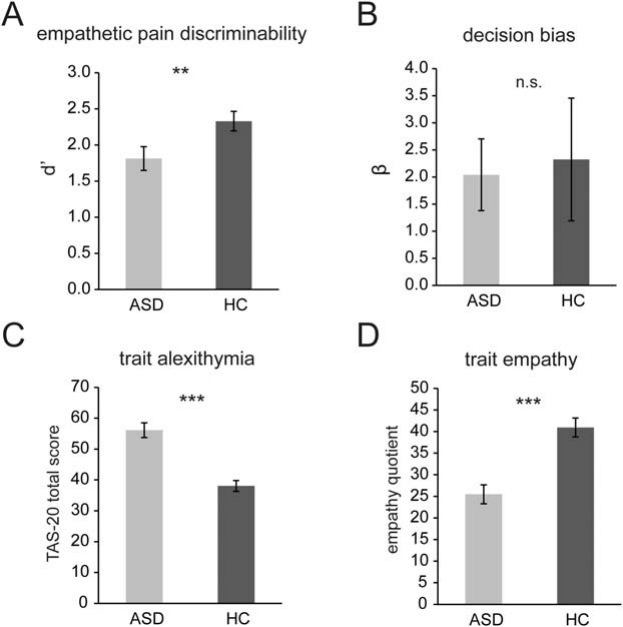
Behavioral and trait measurements. (**A**) ASD group showed impaired empathetic pain discriminability *d*′. (**B**) There was no significant group difference in decision bias *β*. (**C**) ASD group showed greater trait alexithymia, that is, greater difficulty in emotional awareness, measured by the 20‐item Toronto Alexithymia Scale (TAS‐20). (**D**) The ASD group also showed impaired trait empathy, measured by the empathy quotient. ASD, autism spectrum disorder; HC, healthy control. **P < 0.01, ***P < 0.001, n.s., not significant. Error bars indicate standard errors of the mean.

The matrix of hit, false alarm, miss, and correct rejection rates are shown in Table 2. The ASD group showed significantly lower *d*′ than the HC group (bootstrapping *P* < 0.01; Fig. 2A), suggesting that ASD individuals had decreased empathetic pain discriminability. Group difference in decision bias *β* did not reach significance (bootstrapping *P* > 0.4; Fig. 2B). The ASD group also showed greater alexithymia (bootstrapping *P* < 0.001; Fig. 2C) and lower trait empathy (bootstrapping *P* < 0.001; Fig. 2D) than the HC group. We also explored the correlation between *d*′ and trait measures. There was a significant negative correlation between *d*′ and trait alexithymia measured by TAS‐20 (*r* = −0.38, *P* = 0.02), suggesting that lower sensitivity to others' pain was related to higher trait alexithymia across both groups. The correlation between *d*′ and the EQ was not significant for both groups combined or either group separately (*r* = 0.2, *P* > 0.1). These results are consistent with previous findings on impaired empathy and emotional awareness in ASD individuals [Baron‐Cohen and Wheelwright, 2004] and also suggest that our visual empathy paradigm was effective in probing the behavioral characteristics related to empathy in ASD.

**Table 2:** Behavioral results (mean ± SD).

	Hit (choose pain when the image is painful)	False alarm (choose pain when the image is nonpainful)	Miss (choose no pain when the image is painful)	Correct rejection (choose no pain when the image is nonpainful)
ASD	0.85 ± 0.13	0.11 ± 0.10	0.15 ± 0.13	0.89 ± 0.10
HC	0.94 ± 0.04	0.07 ± 0.07	0.06 ± 0.04	0.93 ± 0.07

### Skin conductance response

**Figure 3:**
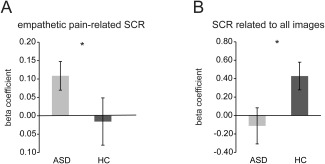
Skin conductance response (SCR). (**A**) ASD group showed increased SCR to painful images compared to HC group. (**B**) SCR related to viewing all images was significantly lower in the ASD group compared to HC group. * *P* < 0.05. ASD, autism spectrum disorder; HC, healthy controls. Error bars indicate standard errors of the mean.

The ASD group showed greater empathetic pain‐related SCR (beta coefficient of regressor “painful images”) compared to the HC group (Fig. 3A; bootstrapping *P* = 0.05). The ASD group's average pain‐related SCRs were greater than zero (one‐sample *t‐*test *t*_(16)_ = 2.79, *P* < 0.05), but the HC group's average pain‐related SCRs were not different from zero (one‐sample *t‐*test *t*_(15)_ = −0.24, *P* > 0.8). These results suggest increased autonomic responses when viewing painful stimuli in the ASD group, but not in the HC group. However, event‐evoked SCRs related to viewing all images (beta coefficient of regressor “all images”) were significantly lower in the ASD group compared to the HC group (Fig. 3B; bootstrapping *P* < 0.05): the ASD participants' overall SCRs were not different from zero (one‐sample *t‐*test *t*_(16)_ = 0.57, *P* > 0.5), and the HC group's SCRs were significantly greater than zero (one‐sample *t‐*test *t*_(15)_ = 2.85, *P* < 0.05). These results are consistent with previous finding of reduced resting state nonspecific SCRs in ASD individuals [Eilam‐Stock et al., 2014].

### fMRI general linear modeling results

**Figure 4:**
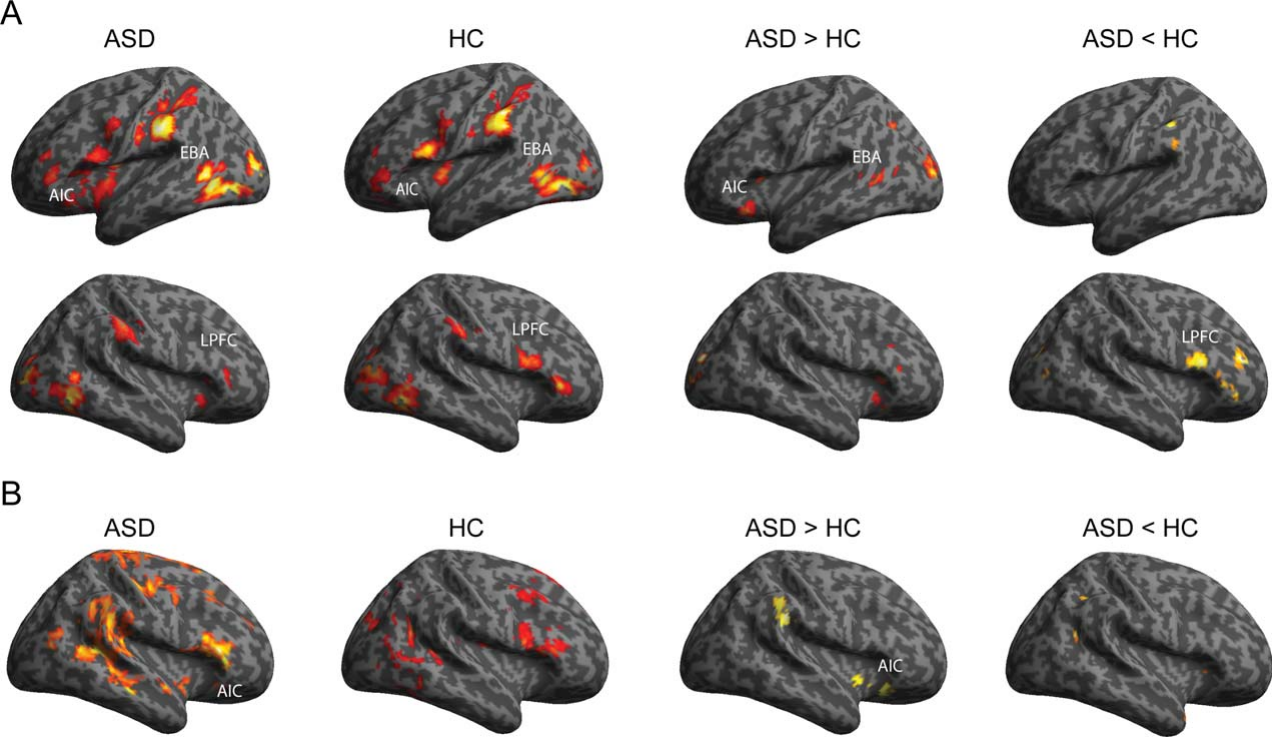
(**A**) Whole brain activations related to empathetic pain. Both ASD and HC groups showed activation in anterior insular cortex (AIC), extrastriate body area (EBA), and lateral prefrontal cortex (LPFC). Adults with ASD showed greater activation in AIC and EBA, and less activation in LPFC compared to HC (see Table III). (**B**) Whole brain activation related to SCR. Compared to HC, ASD individuals showed greater activation in the AIC and several other brain regions (see Table IV). ASD, autism spectrum disorder; HC, healthy control (*P* < 0.05 uncorrected and *k* > 120, equivalent to *P* < 0.05 corrected for multiple comparisons).

The main fMRI GLM analysis revealed that both ASD and HC groups showed empathetic pain‐related activations in left AIC, left EBA, and right LPFC (Fig. 4A and Table 3). These results are consistent with our previous study using the same stimuli [Gu et al., 2010]. Whole brain group comparisons showed that relative to the HC group, the ASD group had greater responses in the AIC and EBA, yet decreased activation of right LPFC (Fig. 4A and Table 3). Additional analyses of brain‐behavior correlation, based on parameter estimates related to “painful images” within the three ROIs, showed that there was no significant correlation between any of the ROIs responses and d′, EQ, or TAS‐20 (all *r* < 0.3, *P* > 0.1). Overall activations related to viewing all images are listed in Supporting Information Table S1.

**Table 3:** Brain activations related to empathetic pain.

Region	L/R	BA	*X*	*y*	*Z*	*Z*	*k*
***Both groups***							
Inferior occipital gyrus^a^	L	19	−46	−70	−4	5.88	1859
Mid occipital gyrus	L	18	−32	−90	6	5.23	
Inferior temporal gyrus	L	20	−46	−46	−14	2.97	
Supramarginal gyrus	L	2	−62	−28	38	5.63	2199
Inferior parietal gyrus	L	40	−34	−42	48	3.91	
Superior parietal gyrus	L	40	−38	−46	58	2.99	
Inferior temporal gyrus	R	37	52	−62	−8	5.2	1624
Mid occipital gyrus	R	18	30	−88	2	4.5	
Mid occipital gyrus	R	19	36	−86	10	4.44	
Rolandic operculum	L	43	−38	−4	14	4.74	3013
Precentral gyrus	L	6	−50	6	22	4.51	
Mid insular cortex	L		−38	0	2	4.09	
Anterior insular cortex*	L		−38	20	2	3.35	
Supramarginal gyrus	R	2	62	−24	40	3.89	596
Supramarginal gyrus	R	40	54	−32	48	2.89	
Supramarginal gyrus	R	40	40	−34	42	2.1	
Inferior frontal gyrus	R	45	54	36	0	3.76	222
Mid/inferior frontal gyrus^a^	R	44	58	14	22	2.92	267
Precentral gyrus	R	6	54	10	40	1.89	
***ASD > HC***							
Calcarine cortex	R	18	20	−84	12	4.38	474
Hippocampus	L	34	−24	−26	−4	3.45	1537
Midbrain	L		−6	−28	−20	3.23	
Midbrain	R		10	−26	−18	3.13	
Lingual gyrus	R	17	14	−54	10	3.43	650
Retrosplenial cortex	R	29	12	−42	18	2.72	
Retrosplenial cortex	L	30	−6	−50	6	3.31	1669
Calcarine cortex	L	17	−12	−78	10	3.13	
Mid occipital gyrus	L	18	−24	−92	12	3	
Hippocampus	R	34	32	−36	−4	3	217
Hippocampus	R	34	30	−22	−10	2.46	
Fusiform gyrus	R	37	34	−36	−12	2.02	
Inferior frontal gyrus	R	45	42	28	8	2.83	251
Anterior insular cortex	R		34	28	14	2.46	
Anterior insular cortex	R		30	26	−2	2.16	
Frontal operculum	L	44	−34	22	−14	2.82	277
Anterior insular cortex	L		−38	12	−12	2.65	
Mid temporal gyrus	L	37	−42	−54	2	2.54	183
Mid temporal gyrus	L	21	−48	−50	6	2.32	
Mid temporal gyrus	L	37	−46	−66	12	2.01	
***ASD < HC***							
Cerebellum	L		−16	−58	−40	2.99	120
Cerebellum	L		−26	−48	−34	1.89	
Superior temporal gyrus	R	22	52	−30	8	2.58	251
Superior temporal gyrus	R	41	40	−38	8	1.93	
Mid/inferior frontal gyrus	R	44	60	20	14	2.51	231
Mid/inferior frontal gyrus	R	44	54	18	30	2.43	

*P* < 0.05 uncorrected and *k* > 120 (equivalent to *P* < 0.05 corrected for multiple comparisons).

a Coordinates used for volume of interest definition. BA, Brodmann's areas. L/R, left/right. ASD: autism spectrum disorder; HC: healthy control.

We also examined whole brain activations correlated with SCRs on a trial‐by‐trial basis (Fig. 4B and Table 4). Compared to HC participants, ASD individuals showed greater correlation with SCR in the right AIC, supramarginal gyrus, anterior cingulate cortex, paracentral lobule, and precuneus. These results suggest that ASD participants show an enhanced coupling between SCR and brain activations in the AIC, when viewing other peoples' body parts. This provides direct evidence supporting abnormally high autonomic and brain responses in ASD.

**Table 4:** Brain activations related to SCR.

Region	L/R	BA	*x*	*y*	*z*	*Z*	*k*
***ASD > HC***							
Supramarginal gyrus	R	2	54	−36	34	2.54	226
Insula	R		40	6	−8	2.45	388
Inferior frontal gyrus	R	47	38	22	−12	2.28	
Anterior cingulate	L	32	−8	16	42	2.42	123
Anterior cingulate	L	32	−16	10	42	2.35	
Paracentral lobule	L	4	−6	−30	56	2	142
Precuneus	L	4	−10	−40	68	1.82	
Precuneus	L	5	−10	−48	64	1.81	
***ASD < HC***							
Caudate	L		−26	10	16	2.98	700
Precuneus	L	29	−12	−46	12	3.46	1344
Caudate	L		24	28	2	3.29	518
Caudate	R		22	24	14	3.13	
Caudate	R		14	22	12	3.03	
Cerebellum	R		26	−56	−44	3	259
Cerebellum	R		32	−60	−40	2.56	
Cerebellum	R		14	−64	−40	2.04	
Inferior temporal gyrus	L	20	−54	−16	−26	2.94	182
Mid temporal gyrus	L	20	−52	12	−24	2.57	
Inferior temporal gyrus	L	20	−44	2	−30	2.55	
Fusiform	R	20	34	−4	−30	2.67	211
Superior temporal pole	R	38	40	14	−26	2.47	
Hippocampus	R	20	34	−6	−22	2.43	
Mid temporal gyrus	L	37	−54	−64	14	2.6	205
Angular gyrus	L	39	−52	−68	28	2.26	
Mid occipital gyrus	L	19	−44	−78	14	2.04	
Cerebellum	L		−4	−46	−50	2.57	245
Cerebellum	L		0	−50	−44	2.56	
Fusiform	L	37	−42	−50	−16	2.05	130
Inferior occipital gyrus	L	37	−52	−62	−16	1.99	
Fusiform	L	19	−42	−70	−16	1.97	

*P* < 0.05 uncorrected and *k* > 120 (equivalent to *P* < 0.05 corrected for multiple comparisons). BA, Brodmann's areas. L/R, left/right. ASD: autism spectrum disorder; HC: healthy control.

### fMRI dynamic causal modeling results

**Figure 5:**
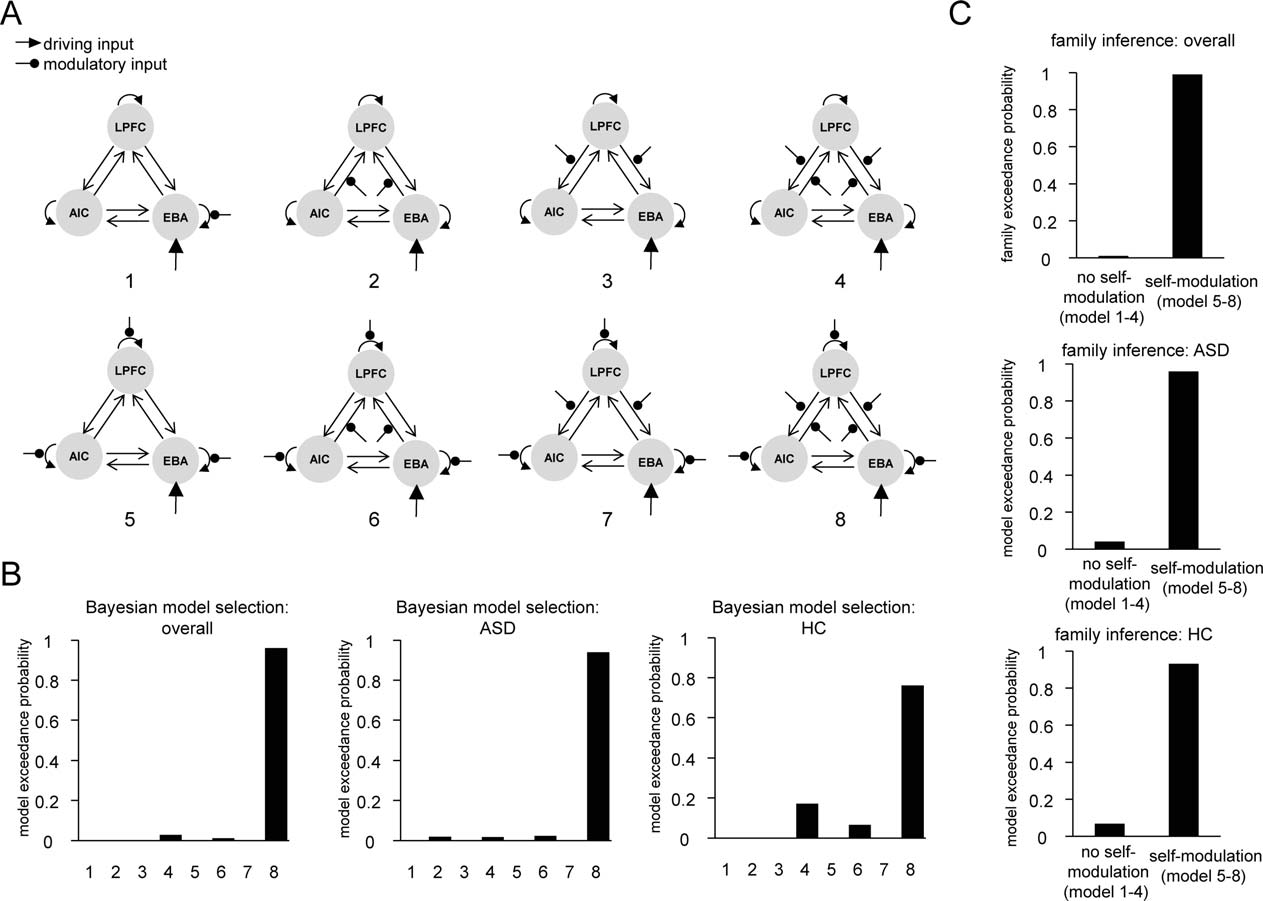
Dynamic causal modeling model specification and comparison. (**A**) Model specification; viewing painful images modulated either no intrinsic connections (Models 1–4), or intrinsic connections of AIC, EBA, and LPFC (Models 5‐8). Additionally, viewing painful images could modulate either no extrinsic connections (Models 1 and 5), forward connections from AIC/EBA to LPFC (Models 2 and 6), backward connections from LPFC to AIC/EBA (Models 3 and 7), or reciprocal connections between AIC/EBA and LPFC (Models 4 and 8). (**B**) Random effect Bayesian model selection (BMS) indicates that Model 8 emerges as the winning model for both groups. (**C**) Random effect BMS at the family level shows that the family of models with modulation of self‐connection by painful images (Models 5‐8) is better than the family of models without such modulation of self‐connection (Models 1‐4). LPFC, lateral prefrontal cortex; AIC, anterior insular cortex; EBA, extrastriate body area; ASD, autism spectrum disorder; HC, healthy control.

We specified eight 3‐region DCMs comprising the AIC, EBA, and LPFC (Fig. 5A). The model with full reciprocal connectivity among these three areas (Model 8) showed the greatest exceedance probability based on model evidence and, therefore, was the best model among all candidate models (Fig. 5B). We also compared models with (Models 5–8) or without (Models 1–4) empathetic pain‐dependent changes in intrinsic connectivity of all three regions at the family level, and the family with intrinsic modulation supervened (Fig. 5C). This family comparison suggests that models where painful images not only modulate inter‐areal connection, but also modulate the self‐connection of each region, were superior to models where there was no modulation of self‐connections. Therefore, the model with full reciprocal connectivity and intrinsic connectivity was the winning model in our defined model space.

**Figure 6:**
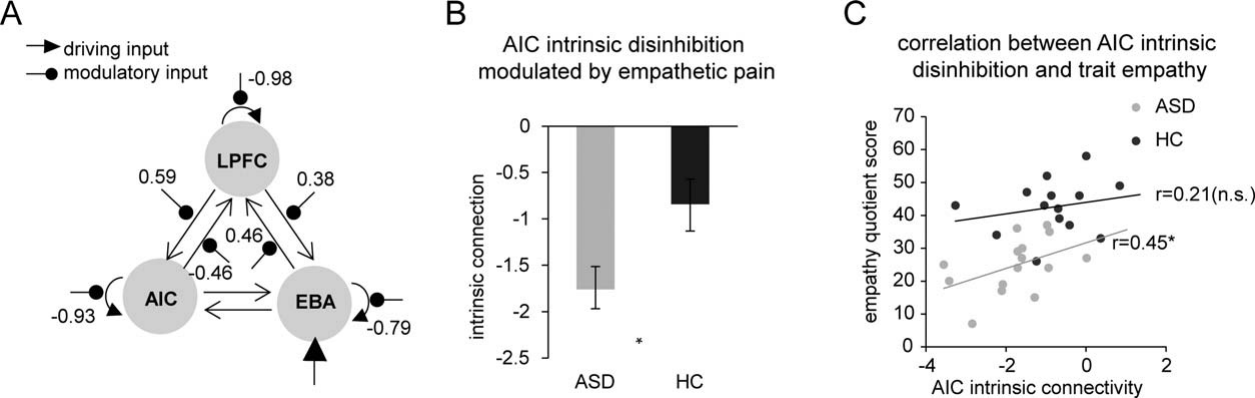
Dynamic causal modeling results. (**A**) The winning model. Numbers represent empathetic pain‐related modulation of connectivity (log‐scale parameters in the leading diagonal of the B connectivity matrix [Friston al., 2003; Penny et al., 2004]. (**B**) ASD group showed greater disinhibition in the AIC modulated by empathetic pain, compared to HC group. (**C**) Greater AIC disinhibition was correlated with less trait empathy measured by the empathy quotient [Baron‐Cohen and Wheelwright, 2004]. * *P* < 0.05 Bonferroni‐corrected for multiple comparisons. n.s., not significant; LPFC, lateral prefrontal cortex; AIC, anterior insular cortex; EBA, extrastriate body area; ASD, autism spectrum disorder; HC, healthy control. Error bars indicate standard errors of the mean.

Figure 6 shows changes in effective connectivity induced by viewing others' pain in the winning model, modeled by the log scale parameters in the leading diagonal of the B connectivity matrix in DCM. In this model, painful images induced disinhibition (i.e., reduced inhibitory self‐connections) in all three regions, as indexed by the negative log‐scale parameters in the B matrix **(**Fig. 6A**)**. Crucially, we found that significant group differences were limited to the modulation of the self‐connection within AIC (Fig. 6B); no significant group differences were detected for pain related changes of other connections (all *P*s > 0.05). As predicted, disinhibition or reduction in the inhibitory self‐connection by pain in the AIC was significantly greater in the ASD group relative to the HC group (Fig. 6B; *P* < 0.05 Bonferroni‐corrected for multiple comparisons). These results suggest that—when viewing another's pain—both ASD and HC groups showed a reduction in AIC self‐inhibition (which explains the increased activation in AIC in the main GLM); however, the ASD group had a significantly greater disinhibition of AIC self‐connection than the HC group. These results provide important evidence for larger empathetic pain‐related AIC disinhibition in adults with ASD, and explain the observed increased AIC activation in ASD participants in this group, when viewing others' pain.

Finally, we explored the relationship between the level of AIC self‐disinhibition and behavioral empathy measurements across participants to confirm the impact of disinhibition, following our hypothesis **(**Fig. 6C). Across groups, greater disinhibition in the AIC correlated with lower levels of trait empathy measured by the EQ (*r* = 0.54, *P* < 0.001), although the correlation only reached significance in the ASD group (*r* = 0.45, *P* < 0.05) and not in the HC group (*r* = 0.21, *P* > 0.2). The group difference in the correlation coefficients between AIC disinhibition and trait empathy was not significant (*z* = 0.65, *P* > 0.5). These results suggest that disinhibition in the AIC might be a contributing factor for impaired trait empathy.

## Discussion

Our study has three main neurophysiological findings. First, ASD participants showed enhanced autonomic signals indexed by SCR when observing others' pain, albeit decreased SCR related to viewing all images of body parts. Second, enhanced SCR was accompanied by increased AIC activation related to empathetic pain in ASD individuals. Third, DCM analysis revealed greater reduction in the negative intrinsic connectivity of the AIC in individual with ASD. Consistent with previous findings [Frith, 1996, 2012; Lord et al., 1994], we also found evidence for impairments in both state and trait empathy in ASD. Specifically, we found reduced empathetic pain discriminability during the empathetic pain task, as well as greater difficulty in emotional awareness and lower empathy levels in self‐report measures in the ASD group. Taken together, these results suggest that abnormally high interoceptive precision, indexed by increased responses at both autonomic and cortical levels, relate to empathy deficits in high‐functioning adults with ASD.

Our finding of abnormal autonomic activity, as indexed by SCR, in the ASD group complements and extends previous findings of sympathetic/parasympathetic imbalance in this disorder [Eilam‐Stock et al., 2014; Hirstein et al., 2001; Kylliainen and Hietanen, 2006]. Specifically, we observed increased pain‐related SCR, yet lower levels of SCR related to watching all images of body parts in participants with ASD. In contrast to earlier studies [Hirstein et al., 2001; Kylliainen and Hietanen, 2006], we used GLM to analyze event‐related SCR in the present study. This is a more sophisticated model than previously used, and is highly suitable for event‐related designs, as it allows the deconvolution of the slow SCR function with the onset of the events [Bach et al., 2010]. Using this model, our findings indicate increased autonomic arousal indexed by SCR when observing others' pain and decreased overall SCR in ASD participants. Therefore, our results suggest a more complex autonomic profile than previously postulated [Eilam‐Stock et al., 2014; Hirstein et al., 2001; Kylliainen and Hietanen, 2006], in which sympathetic activity is decreased at baseline in ASD, with dysregulated, heightened sympathetic responses and arousal when viewing others' pain. These results are consistent with our previous finding of reduced number of nonspecific (nontask‐evoked) SCRs during rest in participants with ASD [Eilam‐Stock et al., 2014].

It is proposed that autonomic responses of bodily states are mapped in the brain in a hierarchical fashion from brainstem and thalamic nuclei, to higher‐order representations in the AIC, anterior cingulate cortex and orbitofrontal cortex, and that these central maps generate the subjective experience of emotions [Craig, 2011; Critchley and Harrison, 2013; Gu et al., 2013a]. It is therefore not surprising that heightened SCR to others' pain were accompanied by increased AIC activation in our ASD participants. Previous findings regarding AIC involvement in empathic processing in individuals with ASD and/or alexithymia (a condition characterized by deficits in emotional awareness that is highly comorbid with ASD) are scarce and mixed, with some demonstrating AIC hypoactivation [Fan et al., 2014], while others demonstrate hyperactivation [Bird et al., 2010; Moriguchi et al., 2007]. We speculate that the differences in findings are due to methodological (e.g., task manipulation) and patient heterogeneity issues. The complex autonomic profile that was observed in our ASD group may also account for the inconsistency in these findings.

Our current finding of significant AIC activation related to empathetic pain is consistent with previous findings on empathy (Corradi‐Dell'Acqua et al., 2011; Gu et al., 2012, 2010; Singer et al., 2009; Wicker et al., 2003] and further suggests that the AIC encodes shared neural representations of subjective (interoceptive) states of self and others. However, it is important to note that abnormally high AIC activation, and accompanied enhanced autonomic signals, could interfere with one's correct behavioral responses to others' pain, as observed in the ASD group. Considering the high level of alexithymia in our ASD participants, it is possible that they had difficulty interpreting their own autonomic response correctly. Previous findings have suggested that the cognitive component of empathy is impaired in ASD [Fan et al., 2014; Hadjikhani et al., 2014; Minio‐Paluello et al., 2009], while emotional contagion may be preserved [Hadjikhani et al., 2014]. Affective arousal during empathic processing, however, as measured by N2 amplitude, was found to be heightened in ASD participants [Fan et al., 2014]. Together with impaired behavioral performance on the empathy‐for‐pain task and reduced trait empathy and emotional awareness, our results provide further support for previous findings and demonstrate both impaired cognitive ability in identifying other's pain (i.e., attenuated empathetic pain discriminability) and heightened affective arousal during empathic processing in ASD. Moreover, because the AIC is important for integrating the emotional and cognitive components of empathy [Gu et al., 2013b], it is also possible that the implicit interoceptive inference is deficient in ASD, and therefore, the AIC is more sensitive to interoceptive cues (prediction errors) due to compensative mechanisms in these individuals.

A handful of studies have directly examined functional connectivity of the AIC in ASD [Ebisch et al., 2011; Eilam‐Stock et al., 2014; Price et al., 2008]. Recently, we demonstrated that SCR was positively correlated with AIC activation in the HC group during rest, while no such correlation was found in the ASD group [Eilam‐Stock et al., 2014]. In addition, AIC functional connectivity was abnormal in the ASD participants [Eilam‐Stock et al., 2014], which is in agreement with findings from other studies demonstrating abnormal activity and connectivity of the AIC in individuals with ASD [Uddin and Menon, 2009; Ebisch et al. 2011]. Functional connectivity during task states, especially effective connectivity during socio‐emotional tasks, is under‐investigated [Uddin and Menon, 2009]. Using DCM, one previous study has examined whether effective brain connectivity was modulated by attention to social and nonsocial stimuli in ASD individuals [Bird et al., 2006]. There was a failure of attention to social stimuli to modulate connectivity between V1 and extrastriate areas. Our DCM result extends these findings on connectivity abnormalities in ASD by suggesting greater reduction in the negative intrinsic connectivity of AIC modulated by empathetic pain, and a direct correlation between such intrinsic disinhibition and trait empathy in ASD.

Importantly, the current findings provide direct support for recent proposals suggesting that failures in Bayesian inference, and particularly aberrant precision (i.e., inverse variance) of the information encoded at various levels of sensorimotor hierarchies, may contribute to socio‐emotional deficits in ASD [Friston et al., 2013b; Lawson et al., 2014; Pellicano and Burr, 2012]. Specifically, abnormally high interoceptive precision (i.e., over reliance on ascending interoceptive information), in the context of interoceptive inference, would result in hypersensitivity of principal AIC neurons that provide downstream predictions of interoceptive signals [Friston, 2010; Seth et al., 2011]. It is this hypersensitivity or increased disinhibition we appeal to explaining the empathetic pain‐related increased AIC neural responses and SCR observed in our study. We suppose that AIC produces interoceptive predictions and updates these predictions based on ascending prediction errors about the physiological states of the body [Craig, 2009; Seth et al., 2011]. AIC also integrates ascending sensory information with the descending top‐down predictions based on multimodal cues that may constitute a sense of a sentient self, subjective awareness, and appropriate bodily responses [Craig, 2009; Gu et al., 2013a]. Crucially, healthy adults are able to attenuate the precision or weight of autonomic concomitants of arousing or salient exteroceptive cues showing others' pain. In adults with ASD, however, there may be a failure to attenuate the influence of autonomic predictions as evidenced by their heightened SCR as well as increased activity and reduced self‐inhibition in the AIC. Thus, abnormally high emotional arousal would result in difficulty in understanding the source of these heightened bodily signals and therefore difficulty in making correct behavioral judgments about emotional stimuli.

Taken together, our results suggest that autonomic and cortical representations of bodily states contribute to high‐level socio‐emotional processes, and that abnormal autonomic and brain activity underlie empathy deficits in ASD. Our results also support the proposal of altered Bayesian inference of bodily and emotional states in ASD.

## Supporting information

Supporting Information
