# Supplementary material for: Autonomic and brain responses associated with empathy deficits in autism spectrum disorder
Source: Hum Brain Mapp. 2015 May 21;36(9):3323–38. doi: 10.1002/hbm.22840 (PMC4545680; doi:10.1002/hbm.22840)
Supplement: Supplementary file 1 — Supporting Information [file HBM-36-3323-s001.docx]

**Supporting Information**

**Figure S1. Alternative whole brain analysis with separate regressors for painful and non-painful images (P<0.05 corrected)**

**
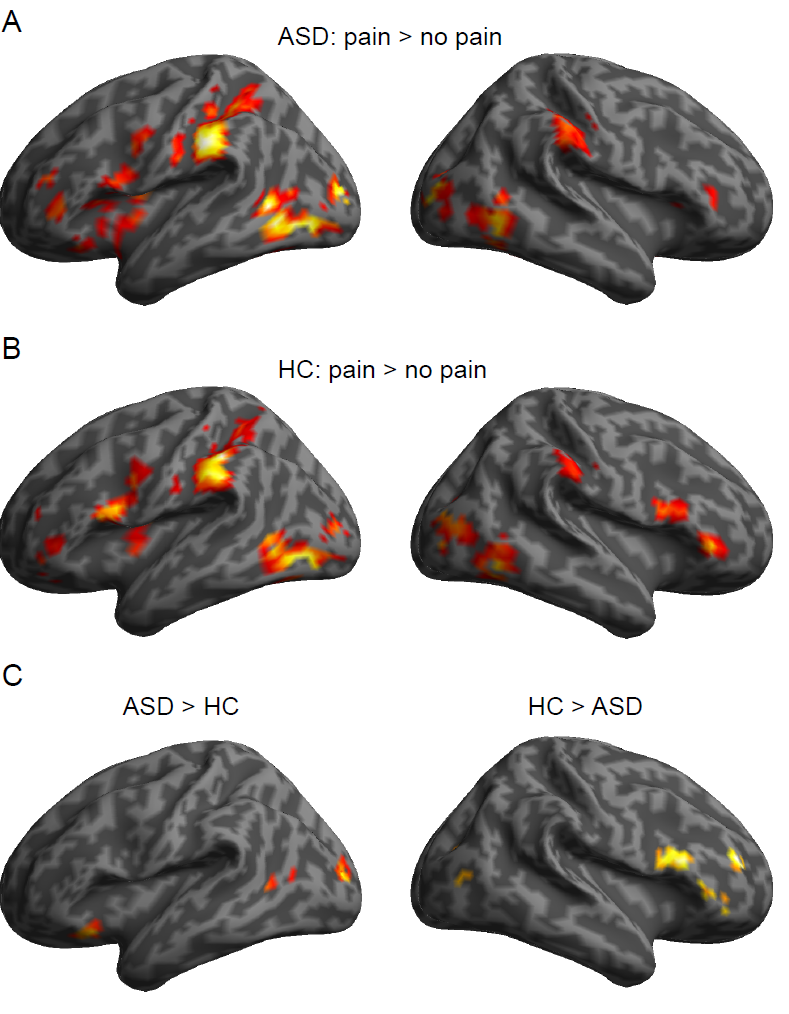
**

**Figure. S2. Monte Carlo simulation of k (cluster size) – p value relationship.**

**
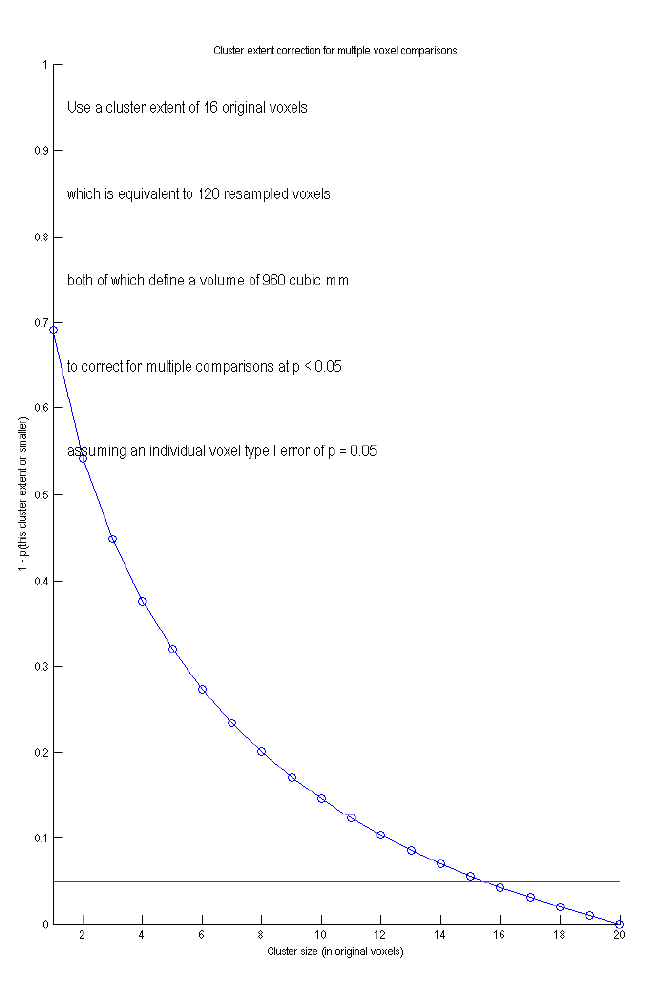
**

**Fig. S3 Illustration of the DCM concept.**

**Table S1.** Overall brain activations related to all stimuli.

| **Region** | **L/R** | **BA** | **x** | **y** | **z** | **Z** | **k** |
| --- | --- | --- | --- | --- | --- | --- | --- |
| ***ASD > HC*** |  |  |  |  |  |  |  |
| Superior frontal gyrus | L | 6 | -20 | -6 | 58 | 3.02 | 439 |
| Precentral gyrus | L | 6 | -20 | -18 | 52 | 2.38 |  |
| Mid frontal gyrus | L | 6 | -28 | 6 | 48 | 2.01 |  |
| Precuneus | R | 19 | 14 | -62 | 46 | 2.23 | 375 |
| Retrosplenial cortex | R | 26 | -8 | -38 | 24 | 2.55 | 262 |
| Retrosplenial cortex | L | 26 | -14 | -44 | 36 | 2.02 |  |
| Mid occipital gyrus | L | 19 | -42 | -74 | 36 | 2.22 | 258 |
| Mid occipital gyrus | R | 19 | -32 | -64 | 24 | 2.14 |  |
| Superior temporal gyrus | R | 22 | 58 | -8 | -6 | 2.36 | 135 |
|  |  |  |  |  |  |  |  |
| ***ASD < HC*** |  |  |  |  |  |  |  |
| Calcarine cortex | R | 18 | 24 | -90 | 2 | 4.21 | 940 |
| Mid occipital gyrus | R | 19 | 30 | -68 | 24 | 2.86 |  |
| Mid occipital gyrus | R | 18 | 24 | -82 | 12 | 2.59 |  |
| Pons | L |  | -8 | -24 | -38 | 3.22 | 1845 |
| Cerebellum | R |  | 22 | -34 | -36 | 3.22 |  |
| Pons | L |  | -12 | -34 | -40 | 3.09 |  |
| Cerebellum | L | 18 | -16 | -70 | -22 | 3.21 | 576 |
| Cerebellum | L | 19 | -24 | -68 | -22 | 2.96 |  |
| Cerebellum | L | 19 | -34 | -66 | -18 | 2.52 |  |
| Mid occipital gyrus | L | 18 | -38 | -84 | 8 | 2.55 | 290 |
| Mid occipital gyrus | L | 18 | -22 | -94 | 4 | 2.45 |  |
| Mid occipital gyrus | L | 19 | -30 | -82 | 14 | 2.09 |  |

*P* < 0.05 uncorrected and k > 120 (equivalent to *P* < 0.05 corrected for multiple comparisons). BA, Brodmann’s areas. L/R, left/right. ASD: autism spectrum disorder; HC: healthy control.
